# Recognizing spatial and temporal clustering patterns of dengue outbreaks in Taiwan

**DOI:** 10.1186/s12879-018-3159-9

**Published:** 2018-06-04

**Authors:** Wei-Ting Lai, Chien-Hsiun Chen, Hsin Hung, Ray-Bing Chen, Sanjay Shete, Chih-Chieh Wu

**Affiliations:** 1 Department of Statistics, College of Management, National Cheng Kung University, Tainan, Taiwan; 2 Institute of Biomedical Sciences, Academia Sinica, Taipei, Taiwan; 3 School of Chinese Medicine, China Medical University, Taichung, Taiwan; 4 Department of Environmental and Occupational Health, College of Medicine, National Cheng Kung University, 1 University Road Tainan, 701 Tainan, Taiwan; 5 Departments of Biostatistics, The University of Texas MD Anderson Cancer Center, Houston, TX USA; 6 Departments of Epidemiology, The University of Texas MD Anderson Cancer Center, Houston, TX USA

**Keywords:** Dengue, Hierarchical, Spatial clustering, Temporal clustering, Zika

## Abstract

**Background:**

Dengue fever is the most common arboviral infection in humans, with viral transmissions occurring in more than 100 countries in tropical regions. A global strategy for dengue prevention and control was established more than 10 years ago. However, the factors that drive the transmission of the dengue virus and subsequent viral infection continue unabated. The largest dengue outbreaks in Taiwan since World War II occurred in two recent successive years: 2014 and 2015.

**Methods:**

We performed a systematic analysis to detect and recognize spatial and temporal clustering patterns of dengue incidence in geographical areas of Taiwan, using the map-based pattern recognition procedure and scan test. Our aim was to recognize geographical heterogeneity patterns of varying dengue incidence intensity and detect hierarchical incidence intensity clusters.

**Results:**

Using the map-based pattern recognition procedure, we identified and delineated two separate hierarchical dengue incidence intensity clusters that comprise multiple mutually adjacent geographical units with high dengue incidence rates. We also found that that dengue incidence tends to peak simultaneously and homogeneously among the neighboring geographic units with high rates in the same cluster.

**Conclusion:**

Beyond significance testing, this study is particularly desired by and useful for health authorities who require optimal characteristics of disease incidence patterns on maps and over time. Among the integrated components for effective prevention and control of dengue and dengue hemorrhagic fever are active surveillance and community-based integrated mosquito control, for which this study provides valuable inferences. Effective dengue prevention and control programs in Taiwan are critical, and have the added benefit of controlling the potential emergence of Zika.

**Electronic supplementary material:**

The online version of this article (10.1186/s12879-018-3159-9) contains supplementary material, which is available to authorized users.

## Background

The global emergence and resurgence of epidemic arboviruses such as dengue and Zika have been dramatic in recent years. Dengue fever is the most common arboviral infection in humans, with viral transmission occurring in more than 100 countries in tropical regions. It is estimated that 390 million dengue infections occur annually, of which 50–100 million cases have apparent clinical manifestations [1–3]. The geographical areas in which transmission of the dengue virus is common have been expanding over the past few decades and all four dengue virus serotypes (DENV1–DENV4) now circulate in Asia, the Americas, and Africa [4]. Compared with other tropical infectious diseases, dengue has a relatively low mortality; however the large scale of human suffering and economic resources used for dengue prevention and control makes it a major global public health problem [1, 5, 6]. There are several factors that contribute to the increased frequency and magnitude of dengue fever and the emergence of dengue hemorrhagic fever, a severe form of the disease. The most important factors are unprecedented growth of human population, unplanned and uncontrolled urbanization, a lack of effective vector control, and globalization [7, 8].

The geographical extension of dengue viral transmission has followed the increased geographic distribution and population densities of *Aedes aegypti*, the principal mosquito vector, which transmits dengue viruses in urban areas of the tropics [7]. Even though great progress has been made in dengue research, particularly in identifying and treating dengue and understanding the structure and replication of the virus, we still do not fully understand why most individuals do not have complications while others experience a severe and fatal hemorrhagic disease. Many unanswered questions remain regarding the virus-host interaction, immune pathology, and influence of genetic variation in the host and virus [9].

Taiwan is infested with both *Ae. aegypti* and *Aedes albopictus* (a secondary mosquito vector), which transmit dengue viruses. The two largest dengue outbreaks in Taiwan since World War II occurred recently, with 15,492 autochthonous cases confirmed in 2014 and 43,419 cases confirmed in 2015. Dengue cases nearly disappeared from the island of Taiwan for 40 years until an outbreak of 4389 cases occurred in 1988. In addition to an outbreak of 5336 cases in 2002, a few small outbreaks occurred between 1989 and 2013. Before World War II, large dengue outbreaks were reported in 1915 and 1931 [10, 11].

Accurately recognizing geographical discrepancies and heterogeneity in dengue incidence patterns and detecting the geographical areas in which the exposure to environmental or viral agents may be responsible for intense dengue incidence will inform disease control and prevention efforts and provide important insights into the etiology of this disease. In this study, we used the map-based pattern recognition procedure and scan test to systematically explore geographical and temporal clustering patterns of dengue incidence in an analysis of Taiwan’s dengue outbreaks in 2014 and 2015. The map-based pattern recognition procedure is designed to recognize hierarchical incidence intensity patterns for some disease over geographical spaces by searching for hierarchical (in intensity) clusters of mutually adjacent areas with high rates [12]. The procedure incorporates information about the intensity rank order into the ordinary adjacency-based test statistic [13], which is designed to analyze data from irregularly arranged and shaped geographic units like the irregular county boundaries within a US state.

Our analysis of the largest Taiwan dengue outbreak in 2015 showed that multiple geographic units with the highest rates of dengue incidence significantly aggregated into 2 separate geographical areas located in Tainan and Kaohsiung in southern Taiwan. More importantly, we determined 3 distinct groups within these geographic units that had the highest dengue incidence rates according to their intensity and delineated 2 separate clusters of hierarchical dengue incidence intensity. Using the scan test, we found that dengue incidence tended to peak simultaneously and homogeneously among the neighboring geographic units with high rates in the same cluster [14].

## Methods

### Study population

Dengue fever is a notifiable communicable disease in Taiwan. Information on dengue cases collected in Taiwan since 1988 is publicly available through the Taiwan Centers for Disease Control (http://www.cdc.gov.tw/english/index.aspx) and the Taiwan Government Open Data website (http://data.gov.tw/en). This information includes the date an individual was diagnosed with dengue infection and his or her residence at diagnosis, place of infection, gender, and age. The study population used for this investigation is patients with laboratory confirmed autochthonous dengue infection, which thus excludes imported cases of dengue. The spectrum of clinical presentations of dengue infection with any one of the 4 viral types is broad. Thus, laboratory confirmation of dengue infection is crucial. Confirmed dengue viral infection in Taiwan is based on a positive diagnosis from any one of 4 laboratory tests: virus isolation, nucleic acid amplification tests, antigen detection, and serological tests. Data on the place where the infection occurred are used in the analysis. If they are unavailable, the individual’s residence at diagnosis is used. Information on the daily local climate variables, including temperature, rainfall, and relative humidity, is available from Taiwan’s Central Weather Bureau (https://www.cwb.gov.tw/eng/index.htm).

Tainan and Kaohsiung are the two largest cities in the southern, tropical region of Taiwan. Kaohsiung is bigger than Tainan in population and area, with 2.78 million residents and 2952 km^2^. Tainan has a population of 1.89 million and 2192 km^2^. *Ae. aegypti*, is dispersed primarily in Tainan, Kaohsiung, and the area to the south of these cities. *Ae. albopictus*, has a widespread distribution throughout most of Taiwan.

**Fig. 1:**
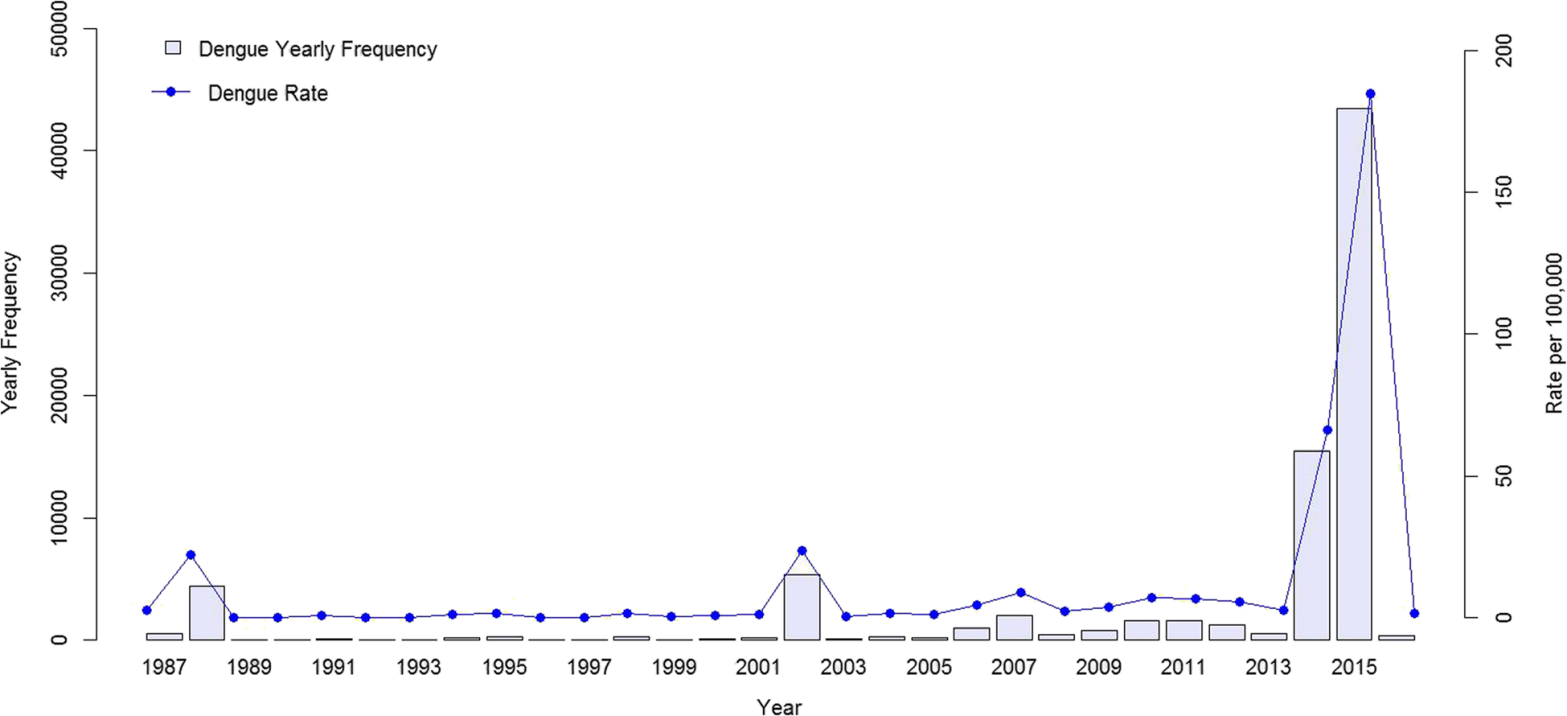
Yearly Frequency Distribution of Confirmed Autochthonous Dengue Cases in 1987–2016

**Fig. 2:**
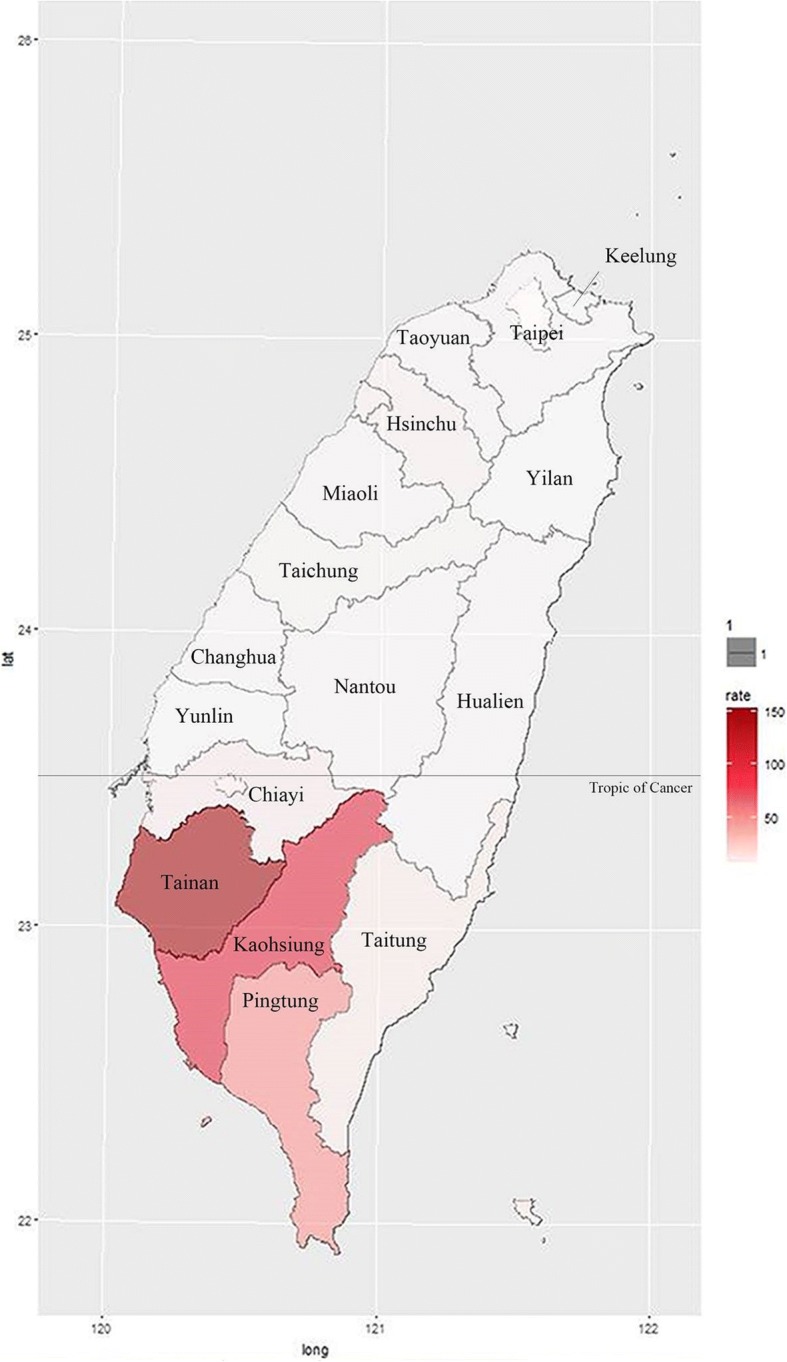
2015 Taiwan Dengue Incidence Intensity Distribution. The figure was generated by the Statistical Package R version 3.3.0 [21]

Figure 1 shows the yearly frequency distribution of confirmed autochthonous dengue cases that occurred between 1987 and 2016. In 2014, there were 15,492 confirmed autochthonous cases in Taiwan, among which 97% (15,034 cases) occurred in Kaohsiung and < 1% (150 cases) occurred in Tainan. In 2015, 98% of the total 43,419 confirmed autochthonous dengue cases occurred in Tainan and Kaohsiung combined, with 22,842 in Tainan and 19,746 in Kaohsiung. The 2015 Taiwan city (and county)-specific dengue incidence intensity distribution is presented in Fig. 2. In 2015, the 3 places with the highest numbers of dengue cases per 100,000 persons were Tainan, with a rate of 1212, Kaohsiung, with 711, and Pingtung County, with 48. All other cities (and counties) had numbers less than 8. In 2016, there were 380 confirmed autochthonous cases in Taiwan.

### Map-based pattern recognition procedure for hierarchical clusters of disease

The method developed by Mantel [15] was generalized by Cliff and Ord, who proposed the test statistic ***B = (1/2) Σ ω***_***ij***_ ***x***_***i***_ ***x***_***j***_ where ***x***_***i***_ ***= 1*** if area ***i*** is a high-risk area for some disease and ***0*** otherwise, and where ***ω***_***ij***_ ***= 1*** if areas ***i*** and ***j*** are mutually adjacent geographically and ***0*** otherwise, ***ω***_***ij***_ ***= ω***_***ji***_, ***ω***_***ii***_ ***= 0*** [16]. The sum ranges over all pairs of areas. It is an adjacency-based test statistic that measures spatial autocorrelation for binary data and uses the distribution of the number of adjacencies of geographic units. When high-risk areas tend to be geographically adjacent to each other, the value of ***B*** tends to be large. Using the test statistic ***B***, one can test the null hypothesis of the random allocation of high-risk areas over the geographical region; that is, high-risk areas do not cluster. Cliff and Ord derived the expressions for the mean and variance of ***B*** under the assumptions of binomial and hypergeometric distributions [13].

Instead of selecting a specific threshold rate of incidence, the map-based pattern recognition procedure proposes to first list the areas under study in rank order based on the disease intensity rates [12]. It starts with classifying the 2 top ranking areas as high-risk areas and calculates the value of ***B***. Subsequently, the procedure includes the area with the 3rd highest rate and the other 2 areas with higher rates as high-risk areas and calculates the corresponding value of ***B***. The *p*-value is the probability that ***B*** is equal to or higher than the observed number of adjacencies involved between these 3 areas with the highest disease intensity rates. The procedure proceeds successively, including exactly one area with high rate according to the rank order and the other areas with higher rates as high-risk areas at each step with the use of ***B***.

Therefore, the procedure provides the *p*-value of ***B*** when the *k* top ranking areas among all areas under study are classified as high-risk areas for each *k* where *k* = 2, 3, 4….. The procedure can classify as many areas as high-risk areas as possible; however, it is unlikely that one would inquire about the possibility of clusters of more than 20% high-risk areas. The main feature of the procedure is to determine the hierarchical incidence intensity pattern through the distribution of *p*-values for *k* = 2, 3, 4…, which will be illustrated in Results.

Instead of relying on the assumptions associated with the asymptotically normal distribution [13], we propose to use simulation-based permutations using 1 million replicates based on the exact district boundary map under study to obtain the null distribution of ***B***. The basic geographic unit used in this report is a “district”, which is the administratively defined subdivision of a city in Taiwan, and which regularly reports health-related information to the city government through its health department. There are 37 and 38 districts in Tainan and Kaohsiung, respectively. The distribution was simulated by randomly selecting exactly *k* districts among the 75 districts of Tainan and Kaohsiung combined 1 million times and counting the number of the adjacent pairs appearing among the *k* districts for each of the 1 million replicates. This process was applied for *k* = 2, 3, 4… 14. Each of the 13 distributions of ***B*** for *k* = 2, 3, 4… 14 is given in Table 1. In this setting, at most 19% (14/75) of the 75 total districts are high-risk districts. With Table 1, we do not require the assumption of asymptotically normal distributions for ***B***. The distribution of ***B*** would closely approximate a Poisson distribution for small values of *k*, because the values of the mean and variance are close, as shown in Table 1.

**Table 1:** Frequency Distributions of the Number of Adjacencies Simulated on the Basis of 1 Million Random Selections in Tainan and Kaohsiung Combined

Test Statistic B																										
	0	1	2	3	4	5	6	7	8	9	10	11	12	13	14	15	16	17	18	19	20	21	22	23	mean	variance
Number of risk districts																										
2	933,817	66,183																							0.066	0.062
3	811,372	179,870	7051	1707																					0.199	0.184
4	654,424	301,846	36,315	6830	464	121																			0.397	0.361
5	487,271	390,927	97,453	20,434	3209	644	52	10																	0.664	0.594
6	335,387	419,120	180,229	50,627	11,572	2565	423	66	10	1															0.994	0.877
7	209,934	387,122	258,564	101,421	31,711	8645	2042	455	85	15	6														1.395	1.215
8	121,202	310,762	301,587	167,122	67,449	22,739	6771	1762	480	98	23	4	1												1.857	1.593
9	63,531	219,245	297,010	224,774	118,538	50,464	18,105	5934	1788	482	99	21	8	1											2.384	2.014
10	30,153	136,056	247,281	252,633	175,657	92,771	41,346	15,969	5630	1762	548	146	37	8	2	1									2.653	2.482
11	12,838	74,469	177,840	239,690	213,280	143,620	78,475	36,271	15,089	5575	1965	652	174	45	15	1	1								3.646	2.985
12	4929	35,989	109,691	191,350	219,975	184,954	124,499	69,938	34,283	14,995	6014	2269	791	237	66	16	4								4.379	3.532
13	1719	15,343	59,144	131,075	189,560	200,876	165,069	111,753	65,553	33,651	15,590	6612	2592	972	340	105	31	13		1	1				5.169	4.108
14	491	5683	27,583	76,015	138,144	181,845	184,653	151,884	105,659	63,626	34,536	16,978	7799	3167	1250	464	142	60	15	4	1	1			6.035	4.721

### Scan test

The scan test is frequently used to detect disease clustering over a temporal series and is structured to test for the largest cluster. The scan test employs a moving window of pre-determined length and finds the maximum number of cases of disease revealed through the window as it slides over the entire period. The scan statistic is the maximum number of events in a window (*t*, *t* + *w*), where *w* is the pre-determined window size as *t* takes on all values in a certain time frame. The model of the scan test that we applied here is based on the assumption of a uniform distribution of events [14]. Here, the scan test was used to test for clustering of dengue incidence and detect the date of the occurrence of maximum dengue incidence in a district.

## Results

### 2015 Tainan and Kaohsiung dengue outbreak

**Fig. 3:**
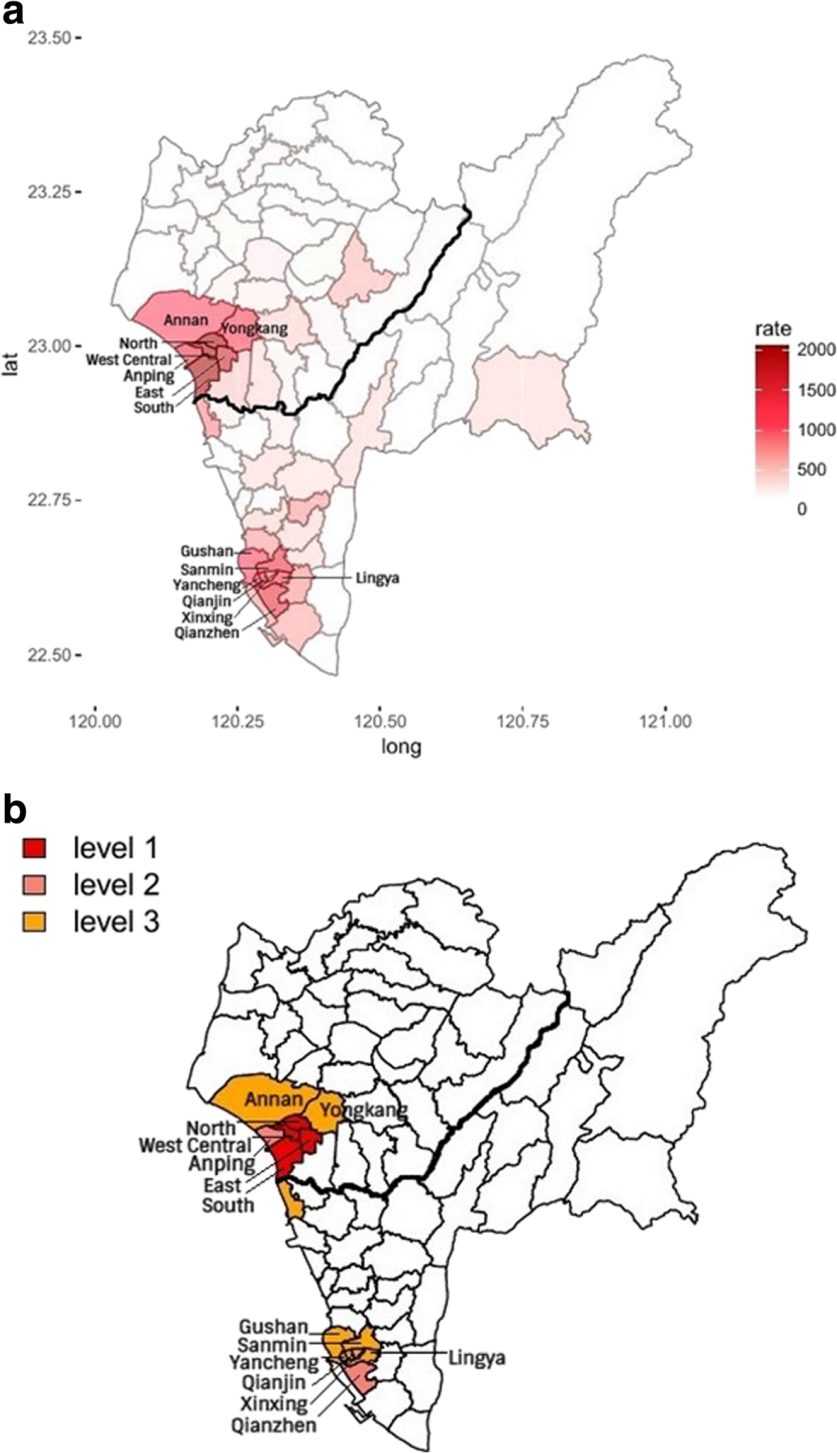
**a**, **b** District-Specific Dengue Incidence **a** Intensity Map and **b** Intensity-Level Map in 2015 in Tainan and Kaohsiung. The figures were generated by the Statistical Package R version 3.3.0 [21]

Tainan and Kaohsiung are geographically adjacent to each other and, therefore, are considered as a single geographical region. The rates, which were the numbers of dengue cases per 100,000 persons, ranged from 0 to 4497 among the 75 districts. Fig. 3a presents the district-specific dengue incidence intensity. We used a cut-off point of 908 in the rates to dichotomize because the difference of 152 between 908 and the next lowest rate of 756 is largest in comparison with the difference between the rates of any other two districts among the upper spectrum of rates, except for the top 5 rates. There were exactly 14 districts with a rate equal to 908 or higher. In this analysis, at most 14 districts with the highest rates were classified as high-risk districts using the map-based pattern recognition procedure. Fifty-five districts or 73% (= 55/75) had rates of 480 or lower.

The 14 top ranking districts are listed by rank according to their rates in Table 2. With each district with a high rate, Table 2 gives the rate, the observed value of ***B*** (= the observed number of adjacencies involved between the district and districts with higher rates), and the *p*-value (determined from the distribution in Table 1). When the (*k* =) 2 top ranking districts in Table 2 (West Central and North districts) are classified as the high-risk districts and the remaining 73 districts are not, the observed value of ***B*** is equal to 1, giving a p-value = *Pr*(***B*** ≥ 1│*k* = 2) = 0.066183 (= 66,183/1 million from Table 1), shown in the 3rd row of Table 2. Table 2 gives the p-value when the *k* top ranking districts are classified as high-risk districts for *k* = 2, 3, 4… 14. Low *p*-values were found for each high-risk district listed in Table 2, except for the North district shown in the 3rd row. The null hypothesis of the randomization of *k* districts with the highest rates was rejected at a nominal significance level of 0.05 for 3 ≤ *k* ≤ 14; that is, the *k* districts with the highest rates showed significant clustering based on ***B***. We also provide the *p*-values in Table 2 based on the Poisson approximation for *k* ≤ 7.

**Table 2:** Cluster Statistic for Districts with the High Rates in 2015 Tainan and Kaohsiung Combined

City^b^	District	Rate^a^	Rank Order	Risk Level	Statistic *B*	*P*-value*	*P*-value**
0	West Central	4497	1	1			
0	North	4313	2	1	1	0.066183	0.063869
0	South	2785	3	1	2	0.008758	0.017360
0	East	1673	4	1	5	0.000121←	0.000059
1	Qianzhen	1415	5	2	5	0.000706	0.000621
0	Anping	1401	6	2	7	0.000077←	0.000080
1	Sanmin	1350	7	3	7	0.000561	0.000610
1	Lingya	1290	8	3	9	0.000126	–
1	Qianjin	1187	9	3	11	0.000030	–
0	Yongkang	1159	10	3	13	0.000011	–
1	Yancheng	1107	11	3	16	0.000001	–
1	Gushan	1000	12	3	18	< 10^−6^	–
0	Annan	984	13	3	22	< 10^−6^	–
1	Xinxing	908	14	3	25	< 10^−6^	–

^a^Number of Dengue Cases per 100,000 Persons

^b^0 indicates Tainan; 1, Kaohsiung

*P-value from Table 1

**P-value using Poisson Approximation

We note that lower p-values of ***B*** indicate high degrees of clustering, which conform to the adjacency-based definition of a cluster [12, 13]. In Table 2, the *p*-values of the 14 high-risk districts appear to be cycling over the rates and are at their relative lowest at the points where the East and Anping districts enter the ranking. We observed a relatively low p-value of 0.000121 when we included the East district (4th in rank) and the other 3 districts with higher rates. In this scenario, the number of high-risk districts was 4 and the observed value of ***B*** was 5, giving a p-value = *Pr*(***B*** ≥ 5│*k* = 4) = 0.000121 (= 121/1 million from Table 1), shown in the 5th row. The p-value jumped to 0.000706 when we included the Qianzhen district (5th in rank) as a high-risk district because the number of high-risk districts became 5 and the observed value of ***B*** remained 5, as shown in the 6th row. The 4 top ranking districts are located in Tainan while the Qianzhen district is in Kaohsiung. The next relatively lower p-value of *Pr*(***B*** ≥ 7│*k* = 6) = 0.000077 (= (66 + 10 + 1)/1 million from Table 1) occurred by including the Anping district (6th in rank) and the other 5 districts with higher rates, leading to the number of high-risk districts = 6 and the observed value of ***B*** = 7, as shown in the 7th row of Table 2.

Correspondingly, we determined the 3 groups of districts to use in constructing hierarchical clusters of mutually neighboring high-risk districts with different levels of intensity using the map-based pattern recognition method [12]. Level-1 districts are the 4 top ranking districts in Table 2 (West Central, North, South, and East districts). Level-2 districts are Qianzhen and Anping, which are respectively the 5th and 6th by rank. Level-3 districts are the 8 districts that rank from 7 to 14. When the level-specific intensity is placed on the map, 2 hierarchical dengue incidence intensity clusters clearly emerge and are located in the urban areas of Tainan and Kaohsiung, respectively, as shown in Fig. 3b. The first cluster geographically expands from the 4 Level-1 districts to 7 mutually adjacent high-risk districts. This geographical area displays the highest dengue incidence intensity, accounting for 50% of dengue cases. In comparison, the second cluster that consists of the other 7 high-risk districts explains 28% of dengue incidence.

The scan test was used not only to determine the clustering of the dengue incidence but also to identify the date of the occurrence of maximum incidence in a high-risk district. Using a window width of 7 days and the time period from week 24 to week 52 of 2015, the scan test was applied to each of the 14 top ranking districts. A very small *p*-value for the scan test was obtained for each of the 7 high-risk districts in Tainan, as shown in Table 3. More importantly, the date of occurrence of the district-specific largest dengue cluster (maximum dengue cases over any 7 consecutive days in a high-risk district) overlapped on 09/18–09/20 among 6 of the 7 adjacent high-risk districts, indicating that the 2015 Tainan dengue outbreak peaked almost simultaneously and homogeneously among the geographically neighboring high-risk districts. A similar phenomenon was observed in Kaohsiung, as shown in Table 3. The peaks of incidence for 5 high-risk districts were during a 2-week period of 11/12–11/25.

**Table 3:** Analysis of Scan Test for Each of 14 Risk Districts in 2015 Tainan and Kaohsiung

District	Rank Order	Total Cases	Statistic of Scan Test	Date	*P*-value
Tainan					
West Central	1	3485	694	9/14–9/20	1.14 × 10^−8^
North	2	5724	891	8/31–9/06	2.23 × 10^−5^
South	3	3502	637	9/15–9/21	2.93 × 10^−7^
East	4	3160	493	9/17–9/23	2.13 × 10^− 5^
Anping	6	916	166	9/14–9/20	3.55 × 10^−7^
Yongkang	10	2675	451	9/18–9/24	2.93 × 10^− 6^
Annan	13	1865	281	9/15–9/21	4.79 × 10^−5^
Kaohsiung					
Qianzhen	5	2726	473	11/13–11/19	1.28 × 10^− 6^
Sanmin	7	4673	596	11/12–11/18	1.01 × 10^−3^
Lingya	8	2251	305	11/13–11/19	3.81 × 10^−4^
Qianjin	9	325	33	09/08–09/14	1.84 × 10^−2^
Yancheng	11	252	30	10/25–10/31	3.33 × 10^−3^
Gushan	12	1369	173	11/14–11/20	1.20 × 10^−3^
Xinxing	14	472	64	11/19–11/25	4.19 × 10^−4^

### 2014 Kaohsiung dengue outbreak

We used the same approaches to study the pattern and distribution of dengue incidence in 2014 Kaohsiung alone. Each of the 6 distributions of ***B*** for *k* = 2, 3… 7 is shown in Additional file 1: Table S1. Correspondingly, Table 4 gives the rate, observed value of ***B***, and p-value (determined from the distribution in Additional file 1:Table S1) for each of the 7 top ranking districts. Small *p*-values were found by including 4 or more districts with the highest rates. These 7 top ranking districts account for 81% of the 2014 Kaohsiung dengue incidence. The weekly distributions of the frequency and rate in 2014 and 2015 in Kaohsiung are given in Additional file 2: Figure S1A. Applying the scan test to each of the 7 high-risk district in the same setting, Additional file 1: Table S2 presents the results, indicating that 4 of the 7 high-risk districts had the date of occurrence of the district-specific largest dengue cluster during a period of 10/15–10/25.

**Table 4:** Cluster Statistic for Districts with the High Rates in 2014 Kaohsiung

District	Rate^a^	Rank Order	Statistic *B*	*P*-value*	*P*-value**
Sanmin	1146	1			
Qianzhen	1029	2	0	1	1
Xinxing	904	3	1	0.320838	0.299527
Lingya	872	4	4	0.004213	0.006101
Xiaogang	729	5	8	0.005743	0.007413
Qianjin	622	6	5	0.000371	–
Fengshan	615	7	11	0.000041	–

^a^Number of Dengue Cases per 100,000 Persons

**P-value from Supplemental Table 1

**P-value using Poisson Approximation

### Effects of temperature, rainfall, and relative humidity

**Fig. 4:**
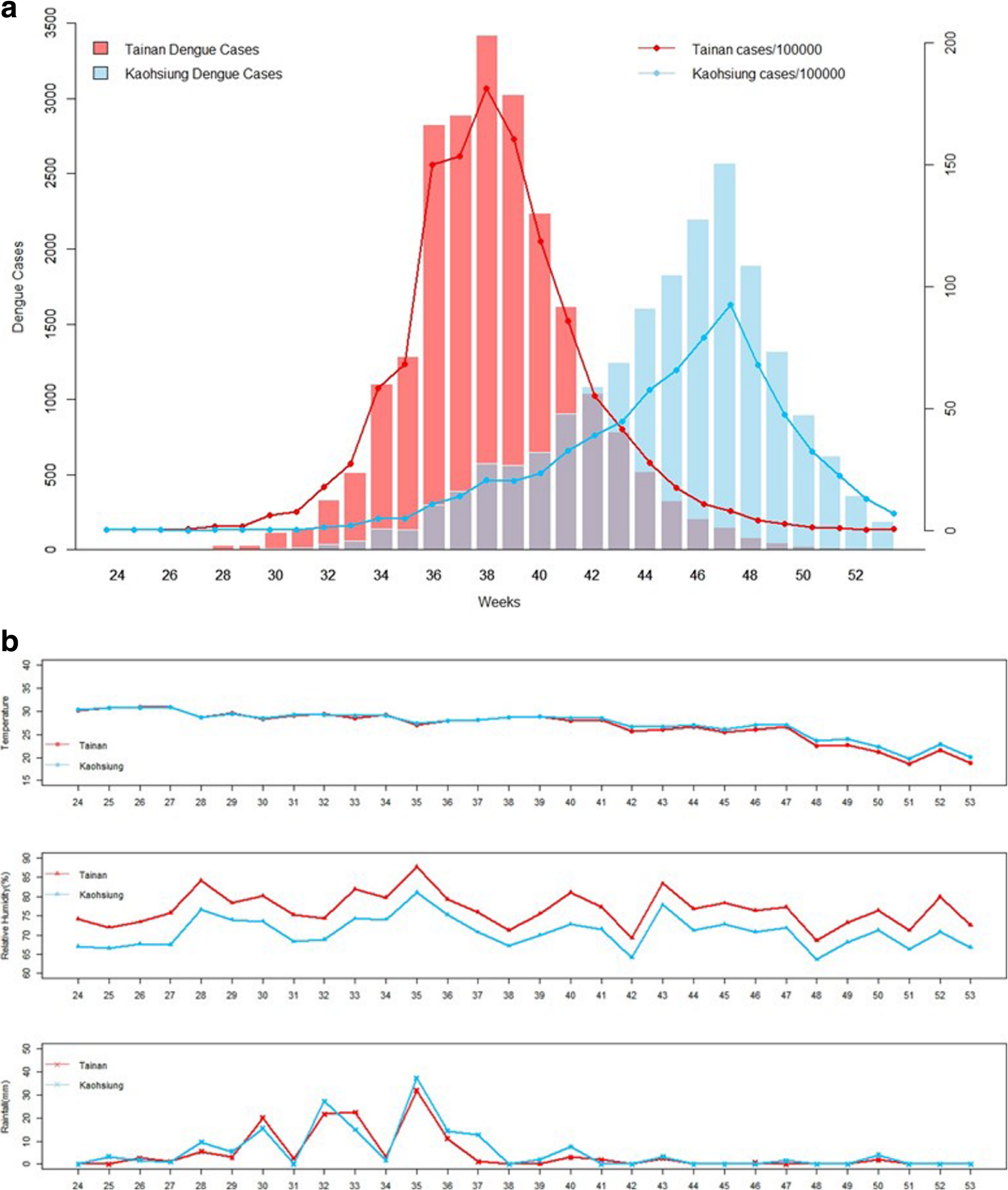
(A, B). **a** Weekly Dengue Incidence Distributions and **b** Weekly Information on Temperature, Relative Humidity, and Rainfall in 2015 Tainan and Kaohsiung

Local weather affects dengue viral transmission and infection [17]. The effects of local climate variables on dengue incidence were considered, including temperature, rainfall, and relative humidity. All 3 local climate variables were similar in Tainan and Kaohsiung. This finding is not surprising as the 2 cities are mutually adjacent. As seen in Fig. 4a, b, relative humidity was consistently higher in Tainan than in Kaohsiung by at most 10% over the outbreak period, and no appreciable difference in temperature and rainfall was observed in 2015. The local weather did not seem to have an effect on the difference in intensity between the 2 distinct hierarchical clusters. The Tainan dengue outbreak peaked on week 38 of 2015, with 3422 dengue cases; the Kaohsiung dengue outbreak peaked on week 47, with 2571 cases. The corresponding rate in Tainan was 181 cases per 100,000 persons, which was nearly twice the rate of 93 cases per 100,000 persons on week 47 in Kaohsiung. While no appreciable change in the local weather between 2014 and 2015 was observed in Kaohsiung, as shown in Additional file 2: Figure S1B, poor environmental management for effective integrated vector controls may be responsible for the worse dengue outbreak and later date of peak incidence occurred in 2015 Kaohsiung.

## Discussion

Historically, Tainan and Kaohsiung experienced the worst dengue incidence in large dengue outbreaks in Taiwan [10, 11]. One major reason is that most areas of Tainan and Kaohsiung are infested with the principal vector, *Ae. aegypti*. In the analysis of the 2015 dengue outbreak, the 4 Level-1 districts had high population density, respectively the 2nd, 1st, 6th, and 3rd by rank in population density in Tainan. This small area experienced extraordinarily high dengue incidence, explaining 37% of the dengue incidence in Tainan and Kaohsiung combined. The 7 districts with the highest dengue incidence rates in 2015 Kaohsiung were also among the districts with the highest population density in Kaohsiung. This indicates that dengue viruses have adapted to the domesticated *Ae. Aegypti* and most transmission occurs in and around the domestic environment in Tainan and Kaohsiung. In addition, it is possible that poor physical environments in Tainan and Kaohsiung could be contributing factors for recent dengue outbreaks and our results of analysis call for better environmental management for integrated vector controls to reduce chance of dengue outbreaks in these regions. We note that the dengue incidence outbreak appeared to be initiated earlier and more concentrated geographically in Tainan than in Kaohsiung, shown in Figs. 4a and 3a, b. They may explain why the dengue outbreak occurred earlier and appeared to rise and fall more rapidly in Tainan. Similar climate might also be yet another reason for the dengue outbreaks in Tainan and Kaohsiung.

Because dengue incidence rates vary substantially by districts and because we attempt to accurately recognize geographical heterogeneity patterns of varying dengue incidence intensity, the map-based pattern recognition procedure is used and provides important epidemiologic pattern analysis. Our investigation exactly delineates the 2 tight clusters, which are distinct in location, intensity, and date of peak incidence. As stressed by Kulldorff (2001), *p*-values should be used as an indicator concerning the evidence for true spatial or space-time clusters rather than maintaining a strict cut-off for the p-value to decide whether to investigate detected clusters or not. The amount of efforts for the investigation should depend on this evidence [18].

A global strategy for dengue prevention and control was established more than 10 years ago, and many efforts have been made to focus on 3 fundamental objectives: surveillance for planning and response, reducing the disease burden and changing behaviors to improve vector control [4]. However, the factors that drive dengue viral transmission and infection continue unabated, and effective vector control remains elusive [6].

In addition, the US Centers for Disease Control (1990) issued a set of guidelines for investigating clusters of health events. According to the guidelines, the four stages are (1) initial contact with and response to the individual who reported the cluster; (2) a preliminary assessment, including evaluations of whether an excess has occurred; (3) a formal feasibility study; and (4) a full etiologic investigation [19]. This study provides valuable information and inference in the second and third stage of the guidelines. We acknowledge some limitations of this study, including (1) this investigation is observational by nature and the exact cause effects cannot be concluded, and (2) the data on the location at which the infection occurred are missing for many individuals, for those the individual’s residence at diagnosis is used in the analysis.

The Zika virus essentially has the same epidemiology and mosquito vectors in urban areas as dengue and is following the same path of global spread via competent mosquito vectors [20]. The potential for the Zika virus to emerge in Taiwan is great due to increased air travel. Thirteen imported cases were reported in Taiwan in 2016.

## Conclusion

Beyond significance testing for disease clustering, our investigation of dengue incidence distribution over spatial and temporal series is desired by and useful for health authorities who require optimal characteristics and patterns of disease incidence on maps and in a temporal series for effective prevention and control programs. Effective prevention and control programs for dengue in Taiwan are critical, and have the added benefit of controlling the potential emergence of Zika.

## Additional files

Additional file 1 **Table S1** Frequency Distributions of the Number of Adjacencies Simulated on the Basis of 1 Million Random Selections in Kaohsiung. **Table S2** Analysis of Scan Test for Each of 7 Risk Districts in 2014 Kaohsiung. (DOCX 17 kb)

Additional file 2 **Figure S1** Weekly Dengue Incidence Distributions and Weekly Information on Temperature, Relative Humidity, and Rainfall in 2014 and 2015 Kaohsiung. (JPG 89 kb)
