# Supplementary material for: Recognizing spatial and temporal clustering patterns of dengue outbreaks in Taiwan
Source: BMC Infect Dis. 2018 Jun 4;18:256. doi: 10.1186/s12879-018-3159-9 (PMC5987425; doi:10.1186/s12879-018-3159-9)
Supplement: Supplementary file 1 — Table S1 Frequency Distributions of the Number of Adjacencies Simulated on the Basis of 1 Million Random Selections in Kaohsiung. Table S2 Analysis of Scan Test for Each of 7 Risk Districts in 2014 Kaohsiung. (DOCX 17 kb) [file 12879_2018_3159_MOESM1_ESM.docx]

**Manuscript Title: Recognizing Spatial and Temporal Clustering Patterns of Dengue Outbreaks in Taiwan**

**Table S1**: Frequency Distributions of the Number of Adjacencies Simulated on the Basis of 1 Million Random Selections in Kaohsiung

| **Test Statistic B** | | | | | | | | | | | | | | | | | |
| --- | --- | --- | --- | --- | --- | --- | --- | --- | --- | --- | --- | --- | --- | --- | --- | --- | --- |
|  | **0** | **1** | **2** | **3** | **4** | **5** | **6** | **7** | **8** | **9** | **10** | **11** | **12** | **13** | **14** | **mean** | **variance** |
| **Number of risk districts** |  |  |  |  |  |  |  |  |  |  |  |  |  |  |  |  |  |
| **2** | 882160 | 117840 |  |  |  |  |  |  |  |  |  |  |  |  |  | 0.118 | 0.104 |
| **3** | 679162 | 291751 | 23319 | 5768 |  |  |  |  |  |  |  |  |  |  |  | 0.356 | 0.310 |
| **4** | 454115 | 414018 | 103037 | 24617 | 3388 | 791 | 34 |  |  |  |  |  |  |  |  | 0.712 | 0.616 |
| **5** | 259424 | 423227 | 220768 | 71478 | 19360 | 4630 | 913 | 188 | 10 | 2 |  |  |  |  |  | 1.187 | 1.010 |
| **6** | 126533 | 329518 | 303807 | 153066 | 60037 | 19529 | 5730 | 1409 | 284 | 73 | 12 | 2 |  |  |  | 1.781 | 1.498 |
| **7** | 51972 | 202033 | 297126 | 233493 | 127722 | 56468 | 21315 | 7057 | 2057 | 583 | 133 | 34 | 6 | 1 |  | 2.491 | 2.054 |
|  |  |  |  |  |  |  |  |  |  |  |  |  |  |  |  |  |  |

**Table S2**: Analysis of Scan Test for Each of 7 Risk Districts in 2014 Kaohsiung

| **District** | **Total Cases** | **Statistic of Scan test** | **Date** | **P-value** |
| --- | --- | --- | --- | --- |
| **Sanmin** | 3985 | 559 | 10/15-10/21 | 2.01×10^-4^ |
| **Qianzhen** | 1994 | 209 | 10/16-10/22 | 1.20×10^-2^ |
| **Xinxing** | 475 | 62 | 10/19-10/25,10/28-11/3 | 7.90×10^-4^ |
| **Lingya** | 1534 | 143 | 10/31-11/6 | 3.43×10^-2^ |
| **Xiaogang** | 1139 | 108 | 10/1-10/7 | 3.02×10^-2^ |
| **Qianjin** | 172 | 19 | 10/15-10/21 | 9.49×10^-3^ |
| **Fengshan** | 2178 | 226 | 11/3-11/9 | 1.32×10^-2^ |
|  |  |  |  |  |
