# Supplementary figures and images for: Recognizing spatial and temporal clustering patterns of dengue outbreaks in Taiwan
Source: BMC Infect Dis. 2018 Jun 4;18:256. doi: 10.1186/s12879-018-3159-9 (PMC5987425; doi:10.1186/s12879-018-3159-9)

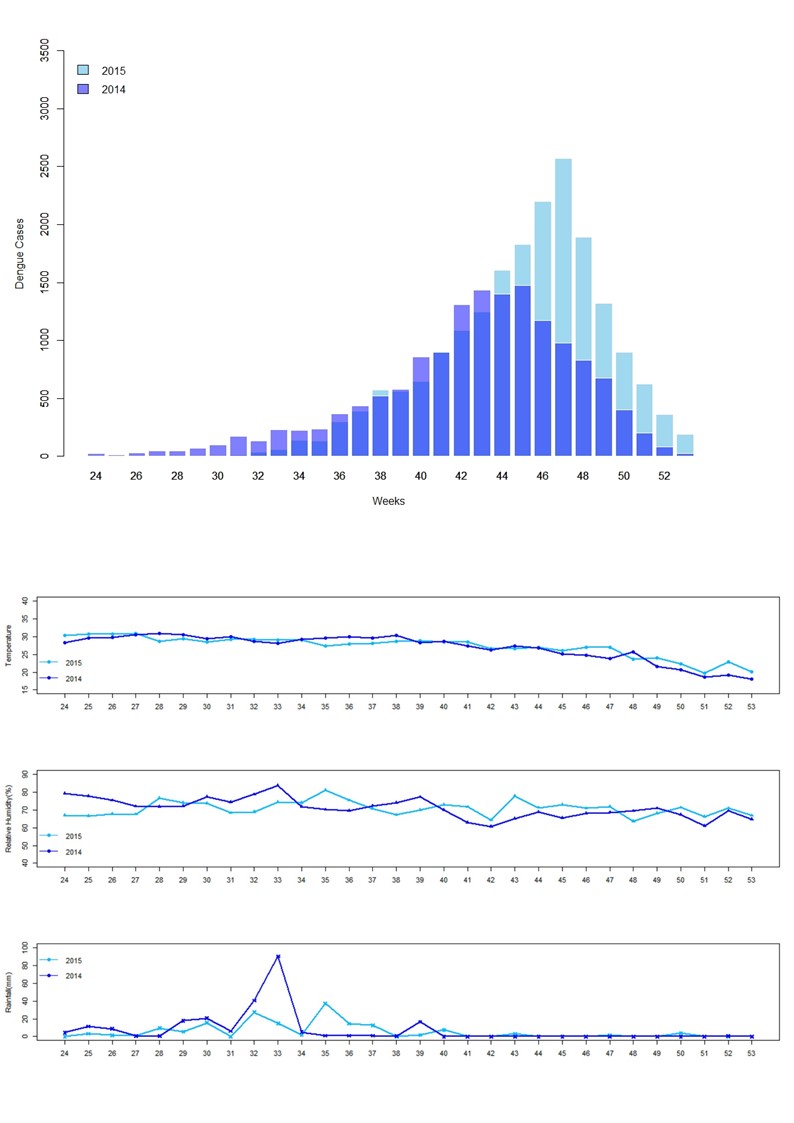

Supplement: Supplementary file 2 — Figure S1 Weekly Dengue Incidence Distributions and Weekly Information on Temperature, Relative Humidity, and Rainfall in 2014 and 2015 Kaohsiung. (JPG 89 kb) [file 12879_2018_3159_MOESM2_ESM.jpg]
